# A set of domain rules and a deep network for protein coreference resolution

**DOI:** 10.1093/database/bay065

**Published:** 2018-07-11

**Authors:** Chen Li, Zhiqiang Rao, Qinghua Zheng, Xiangrong Zhang

**Affiliations:** 1 MOEKLINNS Laboratory, Department of Computer Science and Technology, Xi’an Jiaotong University, 28 Xianning West Road, Xi'an, Shaanxi, PR China; 2 The Key Laboratory of Intelligent Perception and Image Understanding of Ministry of Education, Xidian University, 2 Taibai South Road, P.O. Box 224, Xi'an, China

## Abstract

Current research of bio-text mining mainly focuses on event extractions. Biological networks present much richer and meaningful information to biologists than events. Bio-entity coreference resolution (CR) is a very important method to complete a bio-event’s attributes and interconnect events into bio-networks. Though general CR methods have been studies for a long time, they could not produce a practically useful result when applied to a special domain. Therefore, bio-entity CR needs attention to better assist biological network extraction. In this article, we present two methods for bio-entity CR. The first is a rule-based method, which creates a set of syntactic rules or semantic constraints for CR. It obtains a state-of-the-art performance (an *F*1-score of 62.0%) on the community supported dataset. We also present a machine learning-based method, which takes use of a recurrent neural network model, a long-short term memory network. It automatically learns global discriminative representations of all kinds of coreferences without hand-crafted features. The model outperforms the previously best machine leaning-based method.

## Introduction

**Figure 1.**
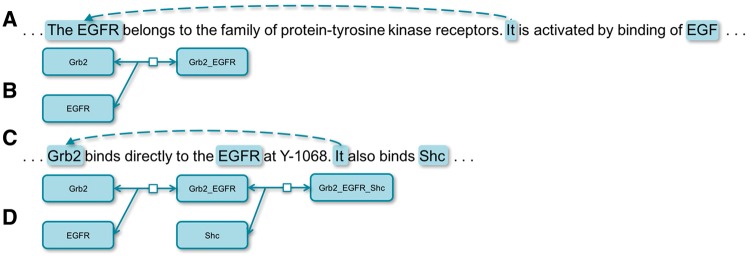
Coreferences in biological texts. **(A)** is a sentence depicting a biological reaction illustrated by (**B** and **C**) is a sentence depicting a biological reaction illustrated by **(D).**

Text-mining techniques have begun to extract bio-events (i.e. reactions) from the scientific literatures in recent years. However, an event at the sentential level is often not capable of depicting a complete bio-reaction. Meanwhile, interconnecting reactions into networks delivers richer and more biologically meaningful knowledge (1). CR (Coreference Resolution) breaks sentential boundaries and connects entities from isolated text units, which is useful for both extracting complete bio-events and constructing bio-networks. For example, it would not be possible to extract the correct event, ‘Grb2 binds EGFR’, from the sentence A in Figure 1 without coreference. Meanwhile, it would not be possible to interconnect two events, ‘Grb2 binds EGFR’ and ‘Grb2 binds Shc’, from the sentence C without CR. Application-wise, CR could be classified into those for general resolution and domain-specific resolution. General CR has been the focus of studies (2–5) while specific domains, such as biomedical entity CR, could well serve particular needs, such as automated extraction of biological networks from Medical Literature Analysis and Retrieval System Online (MEDLINE) (6). In this article, we present two methods of resolving coreferences in bio-texts. One is based on a set of rules, which achieves the state-of-the-art result, and the other is based on a recurrent neural network (RNN) model, which also outperforms the best machine learning-based system. We also try to explore the right situations for using different approaches by comparing two approaches.

### Related work

General CR has a long history of being studied from early rule-based approaches, to machine learning-based methods. Several classic rule-based CR algorithms including the syntax-based Hobbs theory (7), discourse-based centering theory (8) and syntactic knowledge-based RAP algorithm (9). In terms of the strategies of machine learning, the algorithms of CR include four types: mention-pair model (10–12), entity-mention model (13–15), mention-ranking model (2, 16–18) and cluster-ranking model (19–21). In recent years, general CR studies focus on mention-ranking methods. Durrett and Klein (3) proposes a non-linear mention-ranking model that attempted to learn distinct feature representations for anaphoric detection and antecedent ranking by being trained on a pair of corresponding subtasks. Later the model has been extended by incorporating entity-level information produced by a RNN running over the candidate antecedent-cluster (4). Clark and Manning (22) applied reinforcement learning to directly optimize a neural mention-ranking model for coreference evaluation metrics; it was the first time for reinforcement learning being used in CR task.

A few excellent CR systems designed for general domain, such as Stanford (2) and Berkeley (3) CR systems, which are rule-based and machine learning-based, respectively. However, such systems are not efficient while being applied to specific domains, such as biomedical text mining (23). In comparison with general CR, biomedical entity CR starts attracting attentions in recent years due to its great potentials in biological and pharmaceutical research, including the rule-based methods (24–26) and the machine learning-based methods (27–31). BioNLP 2011 Protein Coreference task (32) is a biomedical text-mining task aiming at protein CR. Several systems have been submitted to resolve the problem. Typical systems include Miwa and Thompson (33) using a rule-based method with 55.9% F1-score, which outperforms the others in the task. The best supervised learning method achieves F1 of 34.1% after using four types of features: lexical, proximity, grammatical and semantic (34). D’Souza and Ng (35) later proposed a hybrid approach that combined both learning-based and rule-based method, achieves the state-of-the-art performance with 60.9% F1.

Machine learning-based biomedical entity CR methods mostly utilize mention-pair model, which has the problem of determining the best candidate antecedent. The closest candidate is always chosen as the best answer but it is not proper sometimes. It requires further work to obtain better results on protein CR in order to support other biomedical text-mining tasks more effectively, such as protein–protein interaction extraction.

## Materials and methods

### Protein CR based on syntactic rules and semantic constraints

Domain-specific information could be used as semantic constraints and has been proved to be helpful when applied to protein CR, we explore a new rule-based method to resolve the problem using a set of self-defined syntactic rules and introducing biological semantic constraints. We focus on three types of anaphors, which are relative pronoun, personal pronoun and definite NP(Noun phrase), using different rules. It has been tested on BioNLP corpus and outperforms the best result of the hybrid method.

#### System architecture

**Figure 2.**
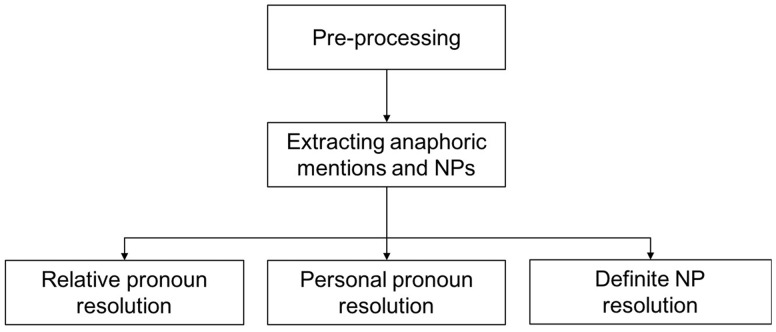
System architecture.

**Figure 3.**
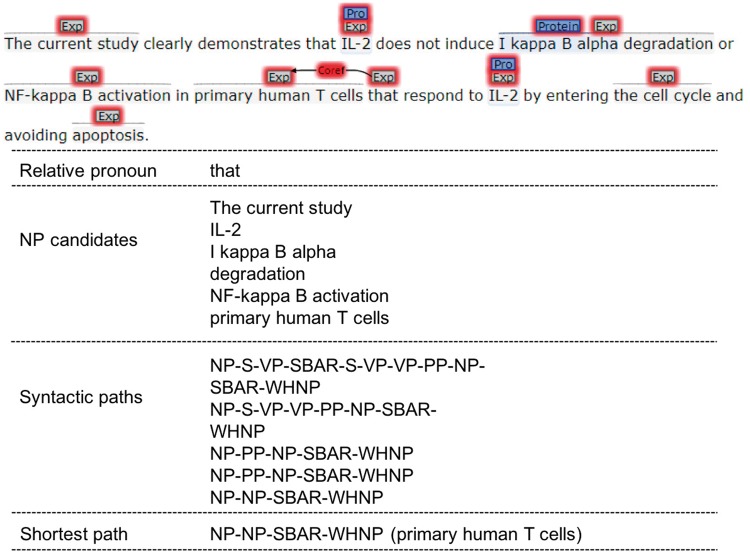
Relative pronoun resolution.

**Figure 4.**
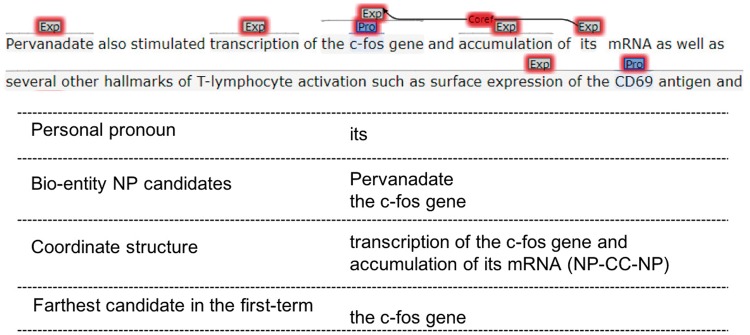
Personal pronoun resolution.

**Figure 5.**
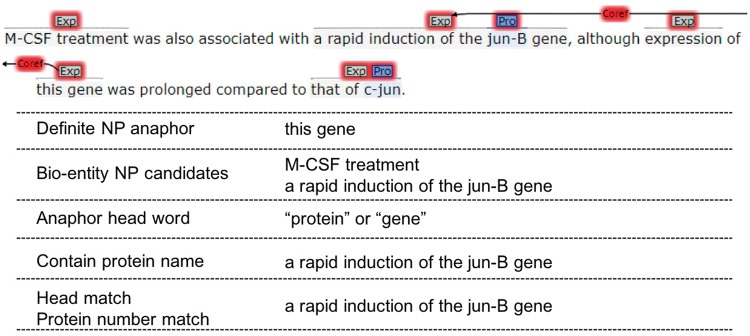
Definite NP resolute.

The proposed system is composed of pre-processing, mention detection and CR. After the pre-processing of the original text, including sentence splitting by Genia Sentence Splitter (36), and tokenization, POS (Part Of Speech) tagging, lemmatization by Stanford CoreNLP (37), and syntactic parsing by Enju Parser (38), three types anaphoric mentions are extracted: relative pronoun (such as which, that), personal pronoun (such as they, its) and definite NP (such as this protein, the gene). According to the statistics of BioNLP Protein Coreference, these three types of anaphors are the most important and have over 95% in quantity (32). And extract NPs to be candidate antecedents; then process the three kinds of coreference relations by either syntactic rules or semantic constraints. Figures 2–5 present the architecture and pipelines of resolution methods.

#### Heuristic-based mention detection

We extract all kinds of mentions from the syntactic tree according to the POS tags. For personal pronoun, we only keep third-person pronoun that is likely to indicate to protein entity, and filter pleonastic it (such as it has been …). For definite NP, we only retain the mentions whose head words are ‘protein’, ‘gene’, ‘factor’, ‘element’, ‘receptor’, ‘complex’ and ‘construct’, these words are more likely to be bio-entity anaphoric mentions according to BioNLP training and development data. For candidate antecedents, we filter the NPs that contain clauses, or are surrounded by other larger NPs.

#### Relative pronoun resolution

Relative pronoun anaphor’s antecedent is always in the same sentence and close to its anaphoric mention. For a relative pronoun, we choose all the NPs that locate before it in the same sentence as its candidate antecedents. Then the syntactic parsing paths are extracted between the relative pronoun and candidates based on the sentence’s syntactic parsing tree. The shortest path is calculated, and the NP in the path is taken as the final antecedent of the relative pronoun.

#### Personal pronoun resolution

Personal pronoun anaphor’s antecedent most likely locates in the same or previous sentence. We first search candidate antecedents in the same sentence, if candidate-set is empty, we would re-extract candidates from the previous sentence and find the possible antecedent. Since personal pronouns have to refer to entities, only the bio-entity NP candidates would be retained, bio-entity NP means that NP contains protein entity name or non-protein entity name.

Once the same sentence’s candidate-set exists, the syntactic parsing tree is traveled from bottom to up beginning with the personal pronoun node. If there are coordinate structures, which include coordinate NP, coordinate VP (Verb Phrase) and coordinate clause, the farthest candidate (by word distance) in the first-term sub-structure would be chosen as the personal pronoun’s antecedent. Otherwise, we would find the closest clause or sentence from the tree, and choose the farthest candidate there to be the antecedent.

When the above set is empty, we choose number-agree bio-entity candidates from the previous sentence. Beginning with the last word of the sentence, we search the syntactic parsing tree from bottom to top, and find the closest clause or sentence that contains candidates. Then we just choose the farthest candidate to be the antecedent.

#### Definite NP resolution

Since definite NP anaphors are often far away from their antecedents and there are not distinct connections between them in syntactic structures, we use semantic constraints instead of syntactic rules to resolve the resolution of definite NP anaphors.

Since we only keep the definite NP anaphors whose head words are ‘protein’, ‘gene’, ‘factor’, ‘element’, ‘receptor’, ‘complex’ and ‘construct’, and they have to refer to entities, we choose bio-entity NP candidates with sentence window 2. The following constraints are applied one by one and the closest candidate meeting the constraints is preferred:

*Constraint 1*: If the anaphor is plural and its head word is ‘proteins’ or ‘genes’, then we filter the candidates that do not contain protein entity name, and choose:

- A candidate whose head word is ‘proteins’ or ‘genes’.
- A candidate that contains protein entities more than 1

*Constraint 2*: If the anaphor is plural and its head word is ‘factors’, ‘elements’, ‘receptors’, ‘complexes’ or con‘structs’, we choose:

- A candidate whose head word is same to the anaphor.
- A candidate that contains bio-entities more than 1.
- A candidate that contains protein entities more than 1.

*Constraint 3*: If the anaphor is singular and its head word is ‘protein’ or ‘gene’, then we filter the candidates that do not contain protein entity name, and choose:

- A candidate whose head word is ‘proteins’ or ‘gene’.
- A candidate that contains 1 protein entity.

*Constraint 4*: If the anaphor is singular and its head word is ‘factor’, ‘element’, ‘receptors’, ‘complex’ or ‘construct’, we choose:

- A candidate whose head word is same to the anaphor.
- A candidate that contains 1 bio-entity.
- A candidate that contains 1 protein entity.

### Long-short term memory-based protein CR

According to the existing methods that are designed for protein CR, rule-based methods need to design precise hand-craft patterns, supervised learning methods, such as Support Vector Machine (SVM) classifier. The method also needs plenty of features so that we have to design domain-specific related features in order to obtain good results. It is difficult and time-consuming. So we explore a deep learning method to solve the protein CR task without hand-craft rules and too many features. Because of the advantage of RNN in solving time sequential information, we use one of its efficient variant, a LSTM model associated with word embedding representation and few features.

LSTM is an RNN architecture designed to be better at storing and accessing information than standard RNNs. And also instead of processing different kinds of anaphors by different resolution methods, the LSTM model processes all kinds of anaphors at the same time and learns global discriminative information from sentences automatically.

#### LSTM-Coref model

**Figure 6.**
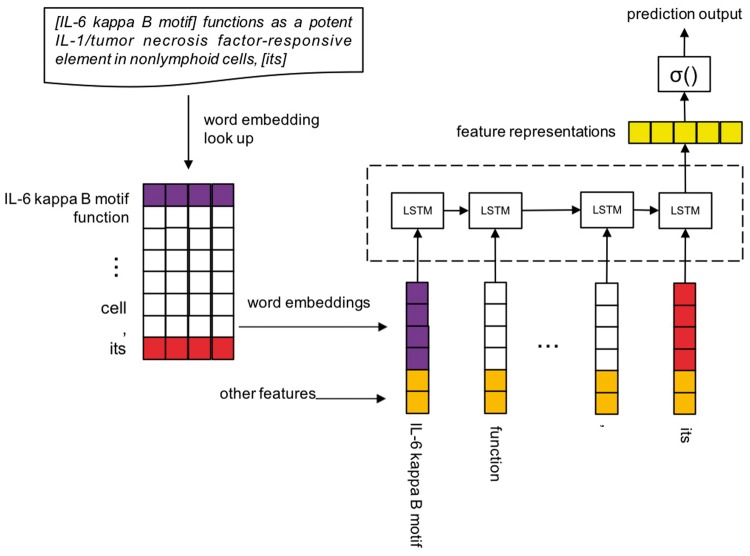
LSTM-Coref.

We formalize the protein CR task as follow. Let $W=w_{1},w_{2},\ldots ,w_{n}$ be a sequence of words that begin with antecedent and end up with anaphor. Also, let $M=m_{1},m_{2},\ldots ,m_{k\;}\;k\;\leq \;n$ be the mentions in this sequence that contain relative pronoun anaphor, personal pronoun anaphor, definite NP anaphor and NP candidate antecedent. All of the four kinds of mentions are extracted by the above syntactic rule method, they could be either a single word or phrase. $m_{1}$ and$\;m_{k}$ is a pair of antecedent and anaphor, $w_{1}$ is the first token of $m_{1}$ and $w_{n}$ is the last token of $m_{k}$. We further assume that $S=s_{1},s_{2},\ldots ,s_{n}$ be the mention-indexes of the words and $s_{j}\in \left\{1,\;2,\ldots k\right\}\cup \{0\}$ where 0 means a word does not match any mention. Finally we replace the words whose mention-indexes are same with their common indexing mention, and get an actual input representation $A=a_{1},a_{2},\ldots ,a_{l\;}\;k\;\leq \;l\leq n$. For example, an instance of *A* may be $m_{1},\;w_{3},\;w_{4},\;m_{2},\;w_{8},\;m_{3},\;w_{11},\;w_{12},\;m_{4}.\;$In this instance $m_{1}$ is the antecedent of the anaphor $m_{4}$. For this task we need to predict the binary label of *A*, it means whether the candidate mention $a_{1}$ is the antecedent of the anaphoric mention $a_{l}$Figure 6 illustrates the architecture of LSTM coreference model.

#### Sentence encoding

In the sentence encoding phrase, we need to transform the representation *A* into a real value vector $X=x_{1},x_{2},\ldots ,x_{l\;}$, and $x_{t}=g(a_{t})$, *g()* is a mapping from a word or mention at to a feature vector $x_{t}$. We take use of two kinds of feature vectors:

- Mention-vector: Instead of using different word embeddings to represent the tokens among a mention, or through an operation on multi-tokens’ word embeddings to obtain a vector to represent a mention, we regard a mention as a whole during the training of Word2Vec (39, 40) model. Only the BioNLP protein CR corpus is used for training the mention-vectors and word vectors, the corpus has been pre-processed, words are replaced by their lemmas and every mention is seen as a whole.
- Other features: Besides the mention-vector, we also use several features that contain important information to help represent mentions. Including:
  Mention type: relative, personal, definite NP, NP;
  Mention number: singular, plural, unknown;
  Mention protein number: 0, 1, >1;
  Mention bio-entity number: 0, 1, >1.

These two feature vectors are concatenated to be real-valued vectors of words or mentions, and then *X* is to be used by LSTM model to learn a more effective representation.

#### LSTM model

Traditional RNN was prone to be the ‘vanishing gradient’ problem. LSTM networks were proposed to alleviate the problem and designed to efficiently learn long-term dependencies. It accomplishes this by keeping an internal state that represents the memory cell of the LSTM neuron. These internal states can only be updated through gates which control the information flowing through the cell state. There are three gates: input, forget and output gate. They are computed as:

(1) $$i_{t}=\sigma (W_{i}\;\cdot \left[h_{t-1},x_{t}\right]+b_{i})$$

(2) $$f_{t}=\sigma (W_{f}\;\cdot \left[h_{t-1},x_{t}\right]+b_{f})$$

(3) $$o_{t}=\sigma (W_{o}\;\cdot \left[h_{t-1},x_{t}\right]+b_{o})$$

The input and forget gate determine the contributions of the current input and the previous output, in the new cell state $c_{t}$. The output gate controls how much of $c_{t}$ is exposed as the output $h_{t}$. They are calculated as:

(4) $$c_{t}=f_{t}*c_{t-1}+i_{t}*tanh(W_{C}\cdot \left[h_{t-1},x_{t}\right]+b_{C}$$

(5) $$h_{t}=o_{t}*tanh(c_{t})$$

We use the output of the last LSTM cell $h_{l}$ as the feature representation of the sequence. Then it is fed into a sigmoid function σ() and produces a probability like output:

(6) $$P\left(Y=1\right)=\sigma (h_{l})$$

Here *Y* is the label of *X*, and *P* (*Y=1*) could be seemed as the probability of existing a coreference relation in A.

#### Training and prediction

We construct coreference pair candidates of different type anaphors by different sentence windows. For relative pronoun, personal pronoun and definite NP anaphor, the window is 0, 1 and 2 separately. For an anaphor, we choose all of the NP antecedent candidates among the corresponding sentence window to construct coreference pair instances. These instances would be used for training the LSTM network or prediction.

We use a binary cross-entropy loss function during the training to optimize the LSTM model:

(7) $$Loss=-\frac{1}{N}\sum_{X}[YlnP\left(Y=1\right)+\left(1-Y\right)ln(1-P\left(Y=1\right))]$$

During the prediction phrase, many NP candidates might be classified as the antecedent of a same anaphor. From them we choose the one who has the maximal probability output to be the final antecedent of the anaphor.

(8) $$Antecedent=\begin{array}{c} \;argmax\;\\ cand\in candidates \end{array}P\left(Y=1|cand,\;Anaphor\right)$$

### Data

BioNLP 2011 protein CR aims at resolving biomedical entities coreference in the scientific literatures, especially specializing in protein and gene coreferences. For example:

*‘*Although it has been previously shown that the [IL-6 kappa B motif] functions as a potent IL-1/tumonecrosis factor-responsive element in nonlymphoid cells, [its] activity was found to be repressed in lymphoid cells such as a Jurkat T-cell line’.

There is a coreference relation between *IL-6 kappa B* motif and *its*. *IL-6 kappa B motif* is an expression that contains protein or gene entity, and *it* is a referring word. In the dataset, anaphors are mainly composed of three type mentions: relative pronoun, personal pronoun, definite NP (Noun Phrase). Antecedents are usually NPs.

General CR is indeed a clustering problem while protein CR is not, the protein CR task demands to find the links that exist coreferene relations in actual semantic expressions. Existing work show that domain-specific information benefits to protein CR and actually by using different methods on different kinds of anaphoric mentions could achieve better results.

## Results

### Protein CR based on syntactic rules and semantic constraints

Table 1 shows the results on BioNLP protein CR test dataset. UU (University of Utah) uses a supervised learning method and has the best result during the tasks: Kim and Tsujii (32). Miwa and Thompson (33) and Nguyen and Kim (41) both use the rule-based methods and obtain better results than the supervised model. D’Souza and Ng (35) process a hybrid approach that combines both rule-based and learning-based method has a superior performance than before. Finally our proposed method that uses syntactic parsing rules and domain-specific bio-rules outperforms all the above results. Out method has the highest recall, and that is the main contribution for improving the performance, in a recall-lower-than-precision state.

**Table 1.** Results on test dataset

	Recall (%)	Precision (%)	*F(%)*
UU	22.2	73.3	34.1
UZ	21.5	55.5	31.0
CU	19.4	63.2	29.7
UT	14.4	67.2	23.8
(41)	52.5	50.2	51.3
(33)	50.4	62.7	55.9
(35)	55.6	67.2	60.9
Proposed	60.2	63.8	62.0

Table 2 presents a detailed comparison between our proposed method and the hybrid method. For relative pronoun resolution, we have exactly the same result, though our method uses syntactic parsing rules while the hybrid method uses a classifier with syntactic path-based features. It is because that compared to other kinds of anaphors, relative pronouns and their antecedents are always in the same sentence and close to each other. For personal pronoun resolution, due to the increase of recall, our method has a great advantage. As said before, it is the most important reason for improving the overall level. For definite NP resolution, it has few quantity and both of us use bio-rules to resolve this type, so we have comparable results.

**Table 2.** Results on development dataset

	(35)	Proposed				
	Recall (%)	Precision (%)	*F* (%)	Recall (%)	Precision (%)	F (%)
Relative pronoun	28.2	83.3	42.2	28.2	83.3	42.2
Personal pronoun	26.3	77.9	39.3	33.6	72.3	45.9
Definite NP	6.9	58.3	12.4	6.9	70.0	12.6
All	59.9	77.7	67.4	68.8	76.0	72.2

### Protein CR based on LSTM

We use BioNLP protein CR training and development dataset to train the LSTM model, and use the test dataset for prediction. The mention-vector is 50 dimensions and obtained by Skip-Gram model using Word2Vec tool. We use one layer LSTM whose hidden units are 200, and the maximal sequential length is 82, which is the maximum from all the training and test sequential instances. We use a maximum of 50 epochs to train the network. The Adam optimizer is applied with batch sizes 80.

Table 3 presents the results on test dataset. When compared with UU’s learning based model that used a SVM classifier with plenty features, our LSTM model with simple features achieves a great advantage on F-score with over 20%. When compared with the two rule-based methods of Miwa and Thompson (33) and Nguyen *et al.* (41), we also have a better result. Although D’Souza and Ng (35)’s work has the best performance, their hybrid approach is not more generalized than ours. They needed to train multi-learning models for different pronouns, and design rules for definite NPs. Although our LSTM model does not distinguish coreference relation types, and learns global feature representations of pronouns or NPs in a same model.

**Table 3.** LSTM-Coref results on test dataset

	Recall (%)	Precision (%)	*F* (%)
UU	22.2	73.3	34.1
(41)	52.5	50.2	51.3
(33)	50.4	62.7	55.9
(35)	55.6	67.2	60.9
LSTM-Coref	54.9	58.0	56.4

To investigate the features used in our LSTM model, we experiment different feature combinations on development dataset. We only use the training dataset to train the model during the experiments. Table 4 presents the results. With merely mention-vector and word vector could generate a significant result that reveals the excellent representation abilities of mention-vector and word vector, but also the strong learning ability of LSTM. What’s more, some other bio related features are contributed to the model’s performance by increasing the recall.

**Table 4.** LSTM-Coref results on development dataset with different feature combinations

	Recall (%)	Precision (%)	*F* (%)
Mention -vec	52.5	65.0	58.1
Mention-vec+features	60.4	61.9	61.2

**Figure 7.**
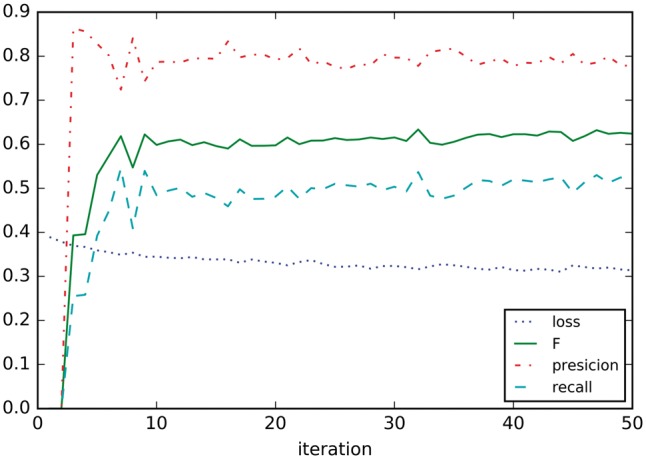
Learning curves on development.

Figure 7 shows the learning curves on development dataset, only the training dataset is used for training the model. It seems that precision, recall and F1 settle after around nine iterations.

### Error analysis

On development dataset, we analyze the experimental results of the two proposed methods from two aspects: missing gold links (MGLs), which are related to recall, and false response links, which are related to precision.

MGLs may be due to three main reasons:

MGL mentions: It happens during the mention detection, and includes both word missing in antecedents and missing of anaphoric mentions.

False links (FLs): It is merely the FLs during the resolution phase in both of the methods. It is because of the lack of rules or performance of learning based models.

Out of range (OOR): It means that a real antecedent exceeds the sentence window we set up.

False gold links may be due to the other three reasons:

Extra links (ELs): A false anaphor does not have coreference relation indeed.

FLs: Same as above.

Beyond mention boundaries (BMB): It happens during the mention detection that covers too many extra words in antecedents.

Tables 5–8 show the detailed error statistics on the development dataset. For MGLs, ‘Others’ stands for the anaphors that do not belong to the three types and would not be dealt with. Such as ‘a transcriptional activator (META)’ and ‘transcriptionally active tetrameric complexes’. From the perspective of anaphor types, DNP (definite NP) is the main reason of MGL errors and personal pronoun is the main reason of spurious gold link errors. Although from the perspective of anaphor types, FLs are the most possible cause of these errors.

**Table 5.** MGLs of rule method

Types	Relative	Personal	DNP	Others	All
MGM	4	2	16	11	**33**
FL	2	9	7	0	18
OOR	0	5	7	0	12
Sum	6	16	**30**	11	63

Bold values are the main errors of coreference types or error types.

**Table 6.** Spurious gold links of rule method

Types	Relative	Personal	DNP	All
EL	6	0	0	6
FL	5	19	6	**30**
BMB	0	8	0	8
Sum	11	**27**	6	44

Bold values are the main errors of coreference types or error types.

**Table 7.** MGLs of LSTM-Coref

Types	Relative	Personal	DNP	Others	All
MGM	7	2	12	11	32
FL	2	14	25	0	**41**
OOR	0	0	7	0	7
Sum	9	16	**44**	11	80

Bold values are the main errors of coreference types or error types.

**Table 8.** Spurious gold links of LSTM-Coref

Types	Relative	Personal	DNP	All
EL	12	0	0	12
FL	8	28	15	**51**
BMB	1	11	0	12
Sum	21	**39**	15	75

Bold values are the main errors of coreference types or error types.

## Conclusion

In this article, we present two methods on protein CR. One is a rule-based method that uses a set of self-defined syntactic rules and semantic constraints. Syntactic rules have been demonstrated to have great potentials on personal pronoun anaphors and it contributes to the whole system by increasing the recall of personal pronoun resolution. The system embodies the proposed outperforms the existing systems and achieves the state-of-the-art result.

The other method is based on LSTM. It does not need hand-crafting rules and features, and is able to learn global discriminative representation features of all kinds of coreferences automatically. The model exceeds other learning-based methods greatly.

## Funding

This work has been supported by National Natural Science Foundation of China (Grant No: 61772409); the Fund of Ministry of Education and China Mobile; ‘The Fundamental Theory and Applications of Big Data with Knowledge Engineering’ under the National Key Research and Development Program of China with grant number 2016YFB1000903; Project of China Knowledge Centre for Engineering Science and Technology; Innovation team of Ministry of Education (IRT_17R86); Innovative Research Group of the Nation Natural Science Foundation of China (61721002); Ministry of Education-Research Foundation of China Mobile Communication Corp (MCM20160404); Professor Chen Li's Recruitment Program for Young Professionals of ‘the Thousand Talents Plan’.

*Conflict of interest*. None declared.
